# Supplementary material for: Transcriptional repression of GTL1 under water‐deficit stress promotes anthocyanin biosynthesis to enhance drought tolerance
Source: Plant Direct. 2024 May 24;8(5):e594. doi: 10.1002/pld3.594 (PMC11117050; doi:10.1002/pld3.594)
Supplement: Supplementary file 3 — Data S3. Protocol for HPLC‐MS polyamine extraction and quantification from A. thaliana leaves. [file PLD3-8-e594-s004.docx]

**Protocol for HPLC-MS polyamine extraction and quantification from *A. thaliana* leaves**

**Preparation**

- 6 N HCl (6 ml of 36.5% HCl + 4 ml water).
- 5% cold PCA (3.213 ml 70% PCA in 41.787 ml HPLC grade water). Keep 5% PCA on ice for at least 30 to make it ice cold.
- Dansyl chloride (7.5 mg/ml of acetone or 78 mg dansyl chloride in 10.4 ml acetone for 24 samples plus 2 extra): make fresh the morning of each dansylation.
- Saturated sodium carbonate (NaCO_3_). Mix as much sodium carbonate in water as possible at room temperature.
- Proline (100 mg/ml water; 300 mg per 3 ml HPLC grade water).
- Dissolution of standards, per [Herrero et al (2016)](https://doi.org/10.1016/j.aca.2016.10.001):
  - Diluted ~30 mg of each standard (including 1,7-diaminoheptane internal standard) in 5 mL 0.1 M hydrochloric acid. Then use this preparation to make 1 mM putrescine, spermine, and spermidine standards (0.25 mM diaminoheptane standard) in 0.4 M perchloric acid. Store at 4°C in the dark.

**Procedure**

1. Crush tissue in liquid nitrogen or use freeze-dried tissue.
2. Add 400 µl 5% cold PCA to 200 µg tissue, then homogenize with plastic pestle and drill for 30 seconds. Swap out pestles after each sample.
3. Add 400 µl 5% cold PCA in homogenized sample and vortex vigorously to homogenize everything in PCA.
4. Incubate samples on ice for 60 min.
5. Centrifuge samples centrifuge at 18,620 g for 30 min at 4°C.
6. Transfer supernatant to 1.5 ml tube and note the volume.
7. Wash the leftover pellet. Add 800 µl 5% cold PCA, vortex it, centrifuge at 12500 g for 20 min and discard the supernatant. Repeat.
8. To suspend the pellet, add 400 µl 5% cold PCA, vortex it and homogenize the pellet into PCA using the pestle and drill setup for 30 seconds.
9. After homogenizing, add 400 µl 5% cold PCA and note the total volume of pellet solution at this step.
10. To quantify conjugated polyamines, transfer 400 µl supernatant into 1 dram glass vials and add 400 µl 6N HCl. Seal the tubes with PTFE caps (it is important to avoid any leakage during hydrolysis).
11. To quantify bound polyamines, transfer 400 µl pellet suspension into 1 dram glass vials and add 400 µl 6N HCl. Seal the tubes with PTFE caps (it is important to avoid any leakage during hydrolysis).
12. Incubate samples at 110℃ for 18 h.
13. After 18 h, cool and measure the volume in each tube by transferring into 1.5 ml tube.
14. Make up the volume to its original value (for example, 600 µl) by adding 5% PCA.

**Dansylation**

1. Take out samples from -20°C if stored.
2. Centrifuge hydrolyzed samples at 18,620 g for 15 min to settle the carbonized material.
3. Transfer 100 µl supernatant (step 8) into glass tubes to dansylate free polyamines. Similarly, transfer 200 µl hydrolyzed and centrifuged supernatant into glass tubes to dansylate conjugated (+free) and bound polyamines (step 18).
4. Add 20 µl 250 µM diaminoheptane (from -20℃) as an internal standard. Remember to label all tubes with different numbers of descriptions to avoid mixing of samples batches.
5. In separate glass tubes, take 15 µl each of three 1 mM polyamines (total 45 µl) and add 75 µl 5% PCA to make up the volume equal to samples containing free polyamines.
   1. This is also the reaction in which standards are set up, either as a mix of the three polyamines, or in three individual reactions (1 for each PA standard). Whether using a mix or individuals (or both) set up five reactions along a concentration gradient so the standard curve is built alongside the sample reactions.
6. Add 200 µl saturated NaCO_3_ (from stirrer) into each sample and standard. In hydrolyzed samples, add NaCO_3_ drop-by-drop to avoid bubbling out due to vigorous chemical reaction.
7. Add 400 µl dansyl chloride and vortex gently.
8. Incubate at 60℃ for 60 min in dark. Set the timer.
9. After 60 min, add 100 µl proline and vortex for 2 seconds.
10. Incubate in a 60℃ water bath for 30 minutes in light.
11. During these 30 min, prepare for the syringes with needles and label new glass tubes.
12. After incubation, add 500 µl toluene and mix well for 30 seconds. Set the timer.
13. Take out approximately 600 µl supernatant in another glass tube and note down the volume. Usually, plant samples will give between 400 µl of usable supernatant before the interface layer starts to interfere. Standard and free samples will give 550 µl.
14. Nitrogen-dry the sample. Keep the nitrogen pressure into tube so much that it should not splash or dry the sample too much.
15. After nitrogen drying, add 150 µl acetonitrile and vortex for 30 seconds.
16. Make up the volume to 250 µl by adding more acetonitrile.
17. Vortex samples for 30 seconds to ensure complete mixing of dansylated polyamines into acetonitrile.
18. Store samples in -20 until further use.
19. Take out tubes and thaw them completely.
20. Add 750 µl of ACN to the dansylated polyamines in acetonitrile, making up a 1 mL total solution.
21. Take the sample in 1ml syringe and filter out.
22. Use 250 µl of the filtered sample in a scintillation vial for HPLC-MS. With a Zorbax SB-C18 5 µm 4.6 x 250 mm column, we used a 25µl injection volume of sample on the column heated to 35˚C over 30 minutes.
