# Supplementary material for: Transcriptional repression of GTL1 under water‐deficit stress promotes anthocyanin biosynthesis to enhance drought tolerance
Source: Plant Direct. 2024 May 24;8(5):e594. doi: 10.1002/pld3.594 (PMC11117050; doi:10.1002/pld3.594)
Supplement: Supplementary file 5 — Data S5. Supporting Information [file PLD3-8-e594-s008.docx]

**Supplemental Figure Captions**

**Supplemental Figure 1.** Average media water content over each irrigation interval of Col-0 and *gtl1-4* plants for the duration of the experiment.

**Supplemental Figure 2.** Stomatal index on the adaxial and abaxial surface in fully expanded well-watered (WW) and water-stressed (WS) Col-0 and *gtl1-4* leaves. Different letters above the bars show statistically significant differences at *P*<0.05 between genotypes and treatment groups within each leaf surface. Error bars represent the standard error, *n=* 8.

**Supplemental Figure 3.** Expression of drought stress response markers in emerging (a) and expanding leaves (b). Data are shown as the log2-transformed normalized expression levels for three biological replicates per genotype in well-watered (WW) and water-stressed (WS) leaves. Statistically significant differences between genotype-treatment combinations are as given for *q*< 0.05 using the EBSeq-HMM package.

**Supplemental Figure 4.** Expression of genes identified by Baerenfaller et al. (2012) as representative of emerging (a) and expanding (b) leaf stages. Data are shown as the log2-transformed normalized expression levels for three biological replicates per genotype-stage. Statistically significant differences between genotype-stage combinations are as given for *q*< 0.05 using the EBSeq-HMM package.

**Supplemental Figure 5.** Expression of cell proliferation stage marker *AN3* as quantified by qPCR (bars ± standard error, left axis) or RNA-Seq (dots, right axis) in well-watered (WW) or water-stressed (WS) Col-0 leaves. Different letters above bars indicate mean separation as tested with EBSeq-HMM. qPCR data was compared against well-watered Col-0, and *** indicates a statistically significant difference at *P*< 0.001. *n=* 3.

**Supplemental Figure 6.** The log2-fold change of gene expression in well-watered (WW) *gtl1-4* relative to WW Col-0 against the FC in water-stressed (WS) Col-0 relative to WW Col-0 and the results of gene ontology overrepresentation analysis in emerging (a–c) and expanding (d–f) leaves. Data are plotted and shown for the 116 and 797 GTL1-primary-regulated genes (Figure 3), representing genes transcriptionally repressed or promoted by GTL1 or water deficit (Q2 and Q4, respectively). In GO analysis plots, all gene categories for which overrepresentation was statistically significant are shown. The vertical line in each plot represents the threshold of the false-discovery rate statistical significance.

**Supplemental Figure 7.** Expression of ribosome biogenesis genes as identified by gene ontology analysis (Fig. 4). Data are shown as the log2-transformed normalized expression levels for three biological replicates per genotype in well-watered (WW) and water-stressed (WS) leaves. Statistically significant differences between genotype-treatment combinations are as given for *q*< 0.05 using the EBSeq-HMM package.
