# Supplementary material for: Transcriptional repression of GTL1 under water‐deficit stress promotes anthocyanin biosynthesis to enhance drought tolerance
Source: Plant Direct. 2024 May 24;8(5):e594. doi: 10.1002/pld3.594 (PMC11117050; doi:10.1002/pld3.594)
Supplement: Supplementary file 6 — Table S1. Accession numbers of genes quantified by qPCR in the current study and their primer sequences. [file PLD3-8-e594-s001.docx]

| **Supplemental Table S1** Accession numbers of genes quantified by qPCR in the current study and their primer sequences. | | | | |
| --- | --- | --- | --- | --- |
|  |  |  |  |  |
|  |  |  |  |  |
| **Gene** |  | **Accession number** |  | **Primer sequence** |
|  |  |  |  |  |
|  |  |  |  |  |
| *GTL1* |  | AT1G33240 |  | F CGTAGGGTTGTTGTTGGCTTA  R GCTGCTGCTCTGCTTGTTACTA |
| *AN3* |  | AT5G28640 |  | F CAGGGAGAAGGAGGAGGTCACAC  R ATTGAAGATCGAGCCGCCATTAG |
| *ACT2* |  | AT3G18780 |  | F AAGTTGTTAGCAGGAGGTCTTGAA |
|  |  |  |  | R CCTGGACCTGCCTCATCATAC |
|  |  |  |  |  |
|  |  |  |  |  |
| All primers are described in the 5’ to 3’ direction | | | | |
