# Supplementary material for: Transcriptional repression of GTL1 under water‐deficit stress promotes anthocyanin biosynthesis to enhance drought tolerance
Source: Plant Direct. 2024 May 24;8(5):e594. doi: 10.1002/pld3.594 (PMC11117050; doi:10.1002/pld3.594)
Supplement: Supplementary file 1 — Data S1. Peer Review [file PLD3-8-e594-s007.pdf]

Plant Direct
Open Access

Transcriptional repression of *GTL1* under water-deficit stress promotes anthocyanin biosynthesis to enhance drought tolerance

Noel Anthony Mano[1,2‡], Mearaj A. Shaikh[2,3], Joshua R. Widhalm[2,3], Chan Yul Yoo[4], Michael V. Mickelbart[1,2,3*]

Decision Letter Round 1:

April 29, 2024
Dr. Michael V. Mickelbart
Purdue University
Horticulture and Landscape Architecture
915 W. State St
West Lafayette, Indiana 47907-2010

MSID: 2024-01484-TW
MS TITLE: Transcriptional repression of GTL1 under water-deficit stress promotes anthocyanin biosynthesis to enhance drought tolerance

Dear Dr. Michael Mickelbart:

I am pleased to inform you that your manuscript "Transcriptional repression of GTL1 under water-deficit stress promotes anthocyanin biosynthesis to enhance drought tolerance" has been accepted for publication in Plant Direct.

Your article will appear online in the next available issue of Plant Direct. To ensure your article gets published as quickly as possible, please pay attention to the steps detailed below. We have found that most of the delays happen at this stage, especially at the payment stage, so please respond as quickly as possible when prompted.

License Agreement: Once your article has been accepted it will move to Production and undergo admin and file checking - you may receive an email with any queries we have at this stage. When all required items are received by the publisher and queries resolved, the corresponding author will receive an email from Wiley's Author Services system which will ask them to log on at https://authorservices.wiley.com/bauthor and will present them with the appropriate license for completion. Your article cannot be published until both the signed

license agreement and payment of the article fee have been received.

Payment of the Open Access Article Publication Fee: All articles published in Plant Direct are fully open access: immediately and freely available to read, download and share. Plant Direct charges a publication fee to cover the publication costs. The corresponding author for this manuscript should have already received a quote from the payments team (cs-openaccess@wiley.com) with the estimated article publication fee; please email cs-openaccess@wiley.com if this has not been received. The corresponding author should log on to the Wiley Author Services site, where the publication fee can be paid by credit card or an invoice. Pro Forma can also be requested. Payment of the publication charge must be received before the article will be published online.

Proofs: You will have the opportunity to look over your paper once more when you receive the author proofs for your article. The proofs will be with you in approximately two weeks. Please note that, in addition to publishing reviewer comments, the author's responses to review comments will also be published alongside the final version of the paper. If you would not like the author's responses to be published, please contact the editorial office at plantdirect@wiley.com .

Promotion of your article: You can help your research get the attention it deserves! Check out Wiley's free Promotion Guide for best-practice recommendations for promoting your work at www.wileyauthors.com/eeo/guide. And learn more about Wiley Editing Services which offers professional video, design, and writing services to create shareable video abstracts, infographics, conference posters, lay summaries, and research news stories for your research at www.wileyauthors.com/eeo/promotion.

Thank you again for your contribution to Plant Direct. If you have any questions, feel free to contact the editorial office at plantdirect@wiley.com .

Sincerely,

Editor, Plant Direct
